# Impact of a vaccination programme in children vaccinated with ProQuad, and ProQuad-specific effectiveness against varicella in the Veneto region of Italy

**DOI:** 10.1186/s12879-018-3017-9

**Published:** 2018-03-05

**Authors:** Carlo Giaquinto, Giovanni Gabutti, Vincenzo Baldo, Marco Villa, Lara Tramontan, Nadia Raccanello, Francesca Russo, Chiara Poma, Antonio Scamarcia, Luigi Cantarutti, Rebecca Lundin, Emilia Perinetti, Xavier Cornen, Stéphane Thomas, Céline Ballandras, Audrey Souverain, Susanne Hartwig

**Affiliations:** 1 Department of Women and Child Health, University of Padova, Padova, Italy; 2 Department of Medical Sciences, University of Ferrara, Ferrara, Italy; 3 Department of Cardiac, Thoracic, and Vascular Sciences, Hygiene and Public Health Unit, University of Padova, Padova, Italy; 4 ATS della Valpadana, Cremona, Italy; 5 Consorzio Arsenàl. IT, Viale Oberdan 5, 31100 Treviso, Italy; 6 Service of Hygiene Promotion and development, Veneto Region, Venice, Italy; 7 Società Servizi Telematici, Pedianet, Padova, Italy; 8 Penta Foundation, Corso Stati Uniti 4, Padova, Italy; 9 MSD Italia S.r.l., Via Vitorchiano, 151 Rome, Italy; 10 Study management, Sanofi Pasteur MSD, 162 avenue Jean Jaurès, Lyon, France; 11 AIXIAL, 4 Rue Barthelemy Danjou, Boulogne-Billancourt, France; 12 Epidemiology, MSD, 162 avenue Jean Jaurès, Lyon, France

**Keywords:** Varicella, Vaccine, Effectiveness, Impact, ProQuad, Italy, Pedianet

## Abstract

**Background:**

Monovalent varicella vaccines have been available in the Veneto Region of Italy since 2004. In 2006, a single vaccine dose was added to the immunisation calendar for children aged 14 months. ProQuad®, a quadrivalent measles-mumps-rubella-varicella vaccine, was introduced in May 2007 and used, among other varicella vaccines, until October 2008. This study aimed to evaluate the effectiveness of a single dose of ProQuad, and the population impact of a vaccination program (VP) against varicella of any severity in children who received a first dose of ProQuad at 14 months of age in the Veneto Region,

**Methods:**

All children born in 2006/2007, i.e., eligible for varicella vaccination after ProQuad was introduced, were retrospectively followed through individual-level data linkage between the Pedianet database (varicella cases) and the Regional Immunization Database (vaccination status). The direct effectiveness of ProQuad was estimated as the incidence rate of varicella in ProQuad-vaccinated children aged < 6 years compared to children with no varicella vaccination from the same birth cohort. The impact of the VP on varicella was measured by comparing children eligible for the VP to an unvaccinated historical cohort from 1997/1998. The vaccine impact measures were: total effect (the combined effect of ProQuad vaccination and being covered by the Veneto VP); indirect effect (the effect of the VP on unvaccinated individuals); and overall effect (the effect of the VP on varicella in the entire population of the Veneto Region, regardless of their vaccination status).

**Results:**

The adjusted direct effectiveness of ProQuad was 94%. The vaccine impact measures total, indirect, and overall effect were 97%, 43%, and 90%, respectively.

**Conclusions:**

These are the first results on the effectiveness and impact of ProQuad against varicella; data confirmed its high effectiveness, based on immunological correlates for protection. Direct effectiveness is our only ProQuad-specific measure; all impact measures refer at least partially to the VP and should be interpreted in the context of high vaccine coverage and the use of various varicella vaccines in this region. The Veneto Region offered a unique opportunity for this study due to an individual data linkage between Pedianet and the Regional Immunization database.

## Background

Varicella-zoster virus is the causal agent of varicella (chickenpox) and herpes zoster (shingles). Varicella is predominantly a childhood disease in unvaccinated populations, with a lifetime risk of acquisition of over 95%. Varicella is highly contagious, with secondary attack rates of 60% to 100% in susceptible individuals [1]. Although varicella is commonly considered a mild disease, it may cause serious complications [2] and represents a sizeable societal burden for patients and their caregivers, sometimes leading to school and work absence [3].

Varicella vaccines have proven to be effective in preventing varicella. Models of the economic impact of childhood varicella vaccination have been performed in Italy, France, and Germany and have projected that routine vaccination programmes could dramatically reduce varicella-related morbidity and lead to a decrease in the number of varicella-related deaths (ranging from 57% to 87% across different strategies and settings) [4, 5]. A vaccine coverage of 90% is projected to lead to savings from both a societal (40% to 60%) and a third-party payer (7% to 61%) perspective [4, 5]. The World Health Organisation advocates routine childhood immunisation against varicella in countries where the disease is an important public health and socioeconomic problem, where the vaccine is affordable, and where high (≥80%) vaccine coverage can be achieved and sustained [6].

In 1974, Takahashi and colleagues developed an attenuated strain of the varicella virus at the University of Osaka, called the OKA strain. Several monovalent and combined live attenuated varicella vaccines that are currently authorised in Europe were derived from this strain. These include the two monovalent vaccines: Varivax® (OKA/Merck strain, Merck & Co., Westpoint, United States) and Varilrix® (OKA/RIT strain, GlaxoSmithKline, Rixensart, Belgium), which were licensed in Europe in the 1990s, and two combined live attenuated quadrivalent vaccines against measles, mumps, rubella, and varicella (MMRV): ProQuad® (OKA/Merck strain, Merck & Co.) and Priorix Tetra® (OKA/RIT strain, GlaxoSmithKline), which were licensed in Europe as from 2006. The quadrivalent vaccines were developed to facilitate childhood vaccination programmes and to support the implementation of routine varicella vaccination.

Clinical trials on ProQuad demonstrated that one dose of the vaccine was immunologically comparable to concomitant administration of the M-M-R®II vaccine (Merck & Co.), a combined measles, mumps, and rubella vaccine, and Varivax [7, 8]. However, there have been no formal studies on the efficacy or effectiveness of ProQuad.

The Veneto Region is located in Northeastern Italy. Monovalent varicella vaccines have been available for private purchase there since 2004, and they were introduced into the vaccination programme (free of charge) in 2006 for children aged 14 months (2005 birth cohort). Catch-up vaccination was also included for those aged 12 years (1994 birth cohort) with no history of varicella [9]. In 2008, the Veneto Region added a second dose of varicella vaccine to the vaccination programme, which is to be administered at 6 years of age.

**Fig. 1:**
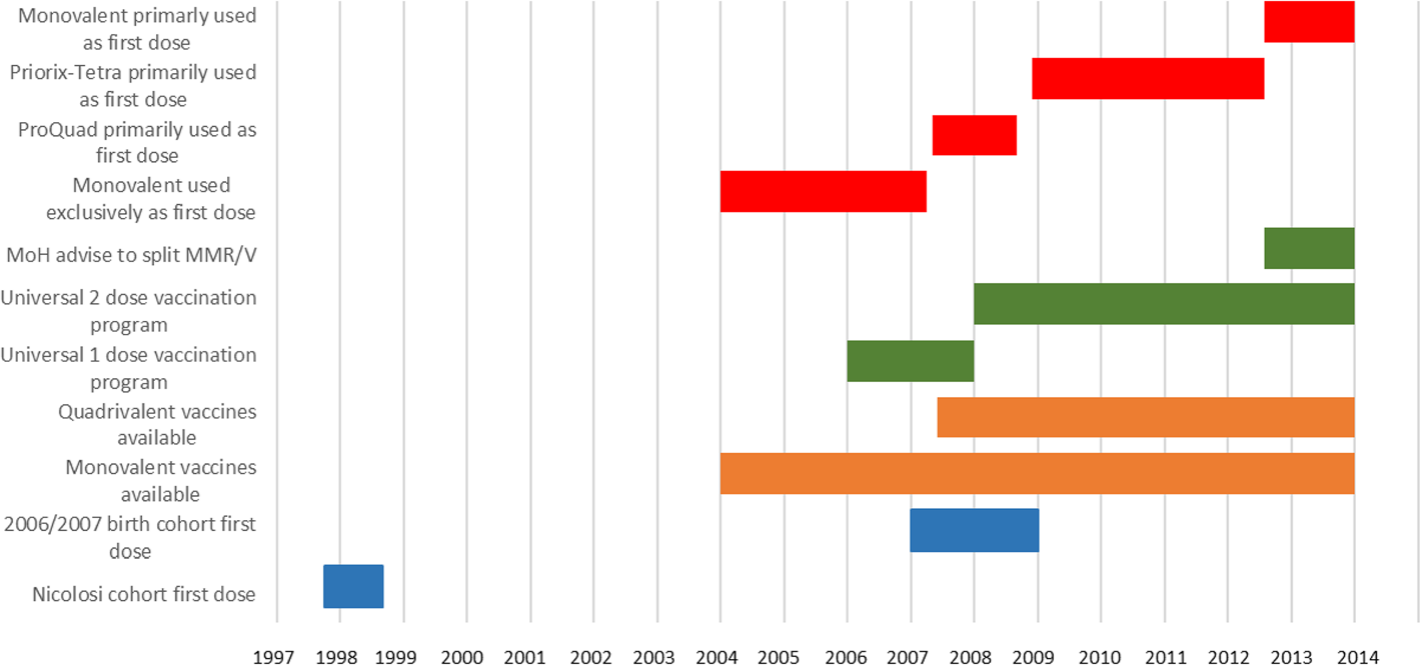
Timeline of varicella vaccine availability and vaccination programs in Veneto region of Italy in relation to study cohort vaccination periods. MoH: Minister of Health; MMR/V: concomitant vaccination with measles, mumps, rubella vaccine and monovalent varicella vaccine

From 2004 to April 2007 in the Veneto Region, a monovalent varicella vaccine, mostly Varivax but also Varilrix, was usually administered concomitantly with the first dose of the measles-mumps-rubella vaccine. In May 2007, the Veneto Region was using ProQuad as a first dose, and in 2008 it began to use it for the second dose as well. However, in October 2008 ProQuad became unavailable and was replaced by the quadrivalent vaccine Priorix Tetra. Monovalent varicella vaccines were also distributed and administered throughout the above-mentioned period (Fig. 1).

In July 2012, the Italian Ministry of Health recommended concomitant injection of a measles-mumps-rubella vaccine and a monovalent varicella vaccine at age 14 months, due to concerns over a small increase in febrile convulsions when injecting MMRV vaccines as a first dose [10]. The use of MMRV vaccines was reserved for the second dose at age 6, where no safety concern had been observed.

The aim of our study was to evaluate the effectiveness of a single dose of ProQuad, and the population impact of a vaccination program against varicella of any severity in children who received a first dose of ProQuad at 14 months of age in the Veneto Region of Italy.

## Methods

### Study setting

The Veneto Region is the fifth-largest region in Italy (after Lombardy, Campania, Lazio, and Sicily) with 21 Local Health Authorities (LHAs) and just over 4.9 million inhabitants. About 700,000 children under 14 years of age live in the Veneto Region, with an annual birth cohort of about 40,000 neonates [11]. After the birth of a child, parents choose a family paediatrician (FP) as an identified primary care provider, as per the requirements of the Italian National Health Service (NHS). It is possible to change FPs, but NHS requirements state that a child must always be registered with one FP. In turn, FPs provide health care services free of charge and are responsible for referring children 0 to 6 years of age to NHS secondary and tertiary care when necessary.

### Data sources

#### Pedianet

**Fig. 2:**
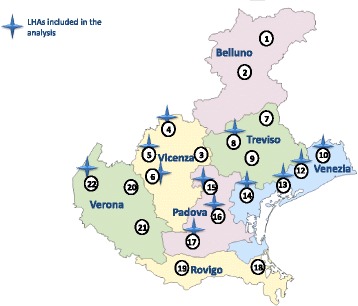
Veneto region with Local Health Authorities (LHAs) that are included in the analysis

In 1999, Pedianet, an independent network of more than 400 FPs, was set up in Italy. Data from children whose parents have signed an informed consent are collected and transmitted to a central Pedianet database in Padova after anonymisation. There are 46 FPs in the Veneto Region that belong to Pedianet, representing about 8% of the paediatricians (*n* = 574) in the region and covering a catchment area of 12 LHAs (Fig. 2). These 46 FPs care for about 52,000 children 0–14 years of age, including an annual birth cohort of 3500 to 4500 neonates, corresponding to about 10% of the regional birth cohort [11].

#### Veneto regional immunization database

The Veneto Regional Immunization Database stores data on all immunisations administered in the region. All paediatric vaccinations included in the Veneto vaccination programme are administered free of charge at the district level by the LHAs. All LHAs record immunisation data using the same software (the Sistema Informativo Anagrafe Vaccinale regionale, SIAVr application), and this data is then sent to the Regional Immunization Database.

### Data collection and linkage

The 46 FPs participating in Pedianet in the Veneto Region identified all children in their patient files who were born in 2006/2007. The Pedianet ID numbers and corresponding fiscal codes (a unique identifier for each person living in Italy) for these children were then encrypted and sent by the FP through a secure channel to the Regional Immunization Database.

Information on the varicella vaccination status of these children and the vaccine brand used was taken from the Regional Immunization Database. Vaccine brand was missing for 67% of the children, as this field was not mandatory until 2010. We extracted information on vaccine type (monovalent or MMRV) whenever possible when information on vaccine brand was missing. As ProQuad was the only MMRV vaccine available in Italy until October 2008, all MMRV vaccines recorded prior this date were attributed to ProQuad. This increased the proportion of vaccines identified as ProQuad to 35%, and decreased the proportion of unknown vaccines to 58%. Vaccination data were then anonymised by discarding the fiscal code and keeping only the Pedianet ID and sent to Arsenàl.IT, Veneto’s Research Centre for eHealth Innovation.

Information on the occurrence of varicella was extracted from the Pedianet database and sent with the Pedianet ID number to Arsenàl.IT, where it was linked to varicella vaccination data. Varicella cases recorded in the Pedianet database are based on physician confirmation only; no laboratory tests were performed.

The study was conducted in accordance with the Declaration of Helsinki, Good Pharmacoepidemiology Practice Guidelines, and local laws, rules, and regulations. The ethics committee of the Azienda Ospedaliera of Padova reviewed the study-related documents and approved the study.

### Study sample

FPs identified 9343 children from the 2006/2007 birth cohort in their records, of whom 1720 were excluded (Table 1). Thus the final number of children in the study sample was 7623:

**Table 1:** Reasons for exclusion

Reasons for exclusion^a^	Number of children
Failure to register in the Pedianet database before 6 months of age	683
No visit to FP in the first year of life	430
< 4 visits to FP in the first 6 years of life	389
Varicella diagnosis in the first year of life	200
Varicella immunisation in the first year of life	26
Received varicella vaccination from FP/missing date of varicella vaccination	16

*FP* family paediatrician

^a^some of the children had more than one reason for exclusion

### Statistical analyses

Children were followed from age 1 year until the occurrence of varicella, until they received the second dose of varicella vaccine (if vaccinated), their 6th birthday, or exit from the Pedianet database, whichever occurred first.

#### Direct effectiveness of a single dose of ProQuad

**Fig. 3:**
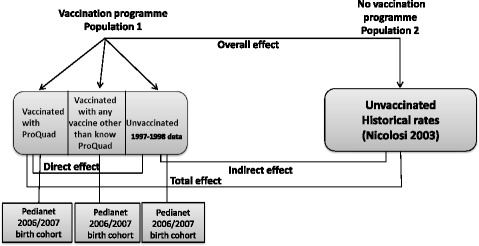
Types of effect and choice of populations

We measured the direct effectiveness (i.e., protection of vaccinee) of a single dose of ProQuad as the relative reduction in the incidence rate of varicella in the 2357 children who received ProQuad as a first dose of varicella vaccine (ProQuad-vaccinated children) in our study sample compared to the 912 unvaccinated children (Fig. 3). All these children belonged to the same population, were exposed to the same vaccination programme, and were followed over the same time period. Direct effectiveness was calculated according to the following formula:

$$ VE=1-\frac{\lambda_v}{\lambda_u}=1- hr $$

where λ_v_ denotes the incidence rate in the ProQuad-vaccinated group, λ_*u*_ the incidence rate in the unvaccinated group and *hr.* the hazard ratio estimated by means of a Cox proportional hazards model with vaccination status as a time-dependent variable. Sex, birth cohort, and number of visits to the FP were tested as covariates. They were entered into the model as strata (not as actual predictors) if they caused the failure of the key assumption of proportionality. To account for the possibility that events within each LHA (or within FPs) are correlated, a Cox proportional hazards model for clustered events was fitted. The vaccination status of each child was set to 0 at the beginning of follow-up and remained at 0 throughout follow-up for unvaccinated children. For vaccinated children vaccination status switched 42 days after vaccination from 0 to 1.

#### Impact of the vaccination programme on varicella in the Veneto region

We assessed three different vaccine impact measures:

1. the total effect, i.e., the combined effect of ProQuad vaccination and being covered by the vaccination programme in the Veneto Region, thus corresponding to the combination of direct and indirect effects.
2. the indirect effect, i.e., the population-level effect of widespread vaccination on unvaccinated individuals in the Veneto Region as a result of reduced transmission.
3. the overall effect, i.e., the effect of the vaccination programme on varicella in the entire population of the Veneto Region, regardless of their varicella vaccination status (Fig. 3) [12].

Data from a historical cohort [13] from the pre-vaccine era was used as the reference population. This cohort consisted of 33,343 children aged 0–14 registered with 35 Pedianet physicians across Italy (with an over-representation of Veneto and Marche regions) between 1 October 1997 and 30 September 1998, of whom 21,783 were estimated to be susceptible to varicella. Incidence rates among unvaccinated subjects from the same historical birth cohort were calculated by dividing reported numbers of varicella by estimated person-years. The person-years were estimated based on the number of susceptible children and varicella cases reported in the historical cohort [13] with assumption of uniform distribution for the onset of the disease, i.e., a child who did not have a varicella diagnosis contributed a full year of exposed person-time, while a child diagnosed with varicella contributed only 6 months of exposed person-time.

##### Total effect

The estimation of the total effect of the vaccination programme in the Veneto Region was evaluated by comparing the observed incidence rate of varicella in the first 6 years of life among the 2357 ProQuad-vaccinated children to corresponding incidence rates from the historical cohort (Fig. 3). It was calculated according to the following formula:

$$ VE=1-\frac{\lambda_v}{\lambda_p}=1- RR $$

Where λ_*v*_ denotes the varicella incidence rate among ProQuad-vaccinated children and λ_*p*_ represents the rate in the unvaccinated historical cohort. The relative risk (RR) denotes the ratio of the varicella incidence rate among ProQuad-vaccinated children to the susceptibility-adjusted incidence rate in the pre-vaccination period [13]. RR and corresponding 95% confidence intervals (CIs) were estimated with the Mantel-Haenszel method for incidence rates. Vaccinated person-time was calculated from date of first varicella vaccination plus 42 days to date of first censoring event, allowing for the delayed onset of immunity after vaccination.

##### Indirect effect

In order to evaluate the indirect effect, the incidence rate of varicella in the 912 unvaccinated children in our study sample was compared to incidence rates from the historical cohort [13] (Fig. 3). The indirect effect was defined as:

$$ VE=1-\frac{\lambda_u}{\lambda_p}=1- RR $$

where λ_u_ denotes the incidence rate in unvaccinated children, λ_p_ the incidence rate in the historical cohort, and RR the incidence rate ratio. RR and corresponding 95% CIs were estimated with the Mantel-Haenszel method for incidence rates. Unvaccinated person-time was calculated from age 1 year until either a first dose of ProQuad or first censoring event, whichever occurred first.

##### Overall effect

In order to evaluate the overall effect, the incidence rate of varicella among all 7623 children (vaccinated and unvaccinated) in our study sample was compared to incidence rates from the historical cohort (Fig. 3) [13]. The overall effect was defined as follows:

$$ VE=1-\frac{\lambda_{overall}}{\lambda_p}=1- RR $$

where λ_overall_ denotes the average incidence rate in the population covered by the vaccination programme, λ_p_ the incidence rate in the historical cohort, and RR the incidence rate ratio. RR and corresponding 95% CIs were estimated with the Mantel-Haenszel method for incidence rates. Person-time was calculated from age 1 year until date of first censoring event.

## Results

**Fig. 4:**
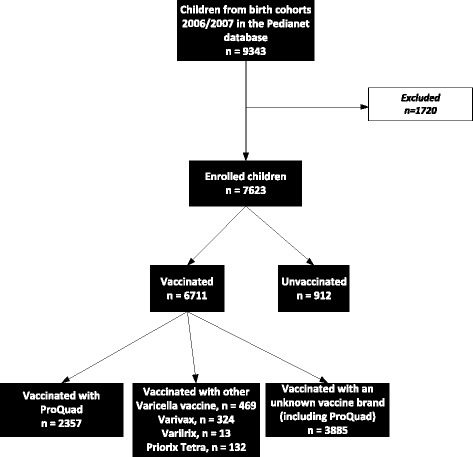
Flow chart

Among the 7623 children in our study sample, 6711 (88%) were vaccinated against varicella. Among vaccinated children, 2357 (35.1%) received ProQuad and 4354 (64.9%) received a different varicella-containing vaccine (Varivax, Varilrix, Priorix Tetra, or an unknown brand). Our unvaccinated population numbered 912 (Fig. 4). We compared sex distribution, birth cohort (2006 and 2007) and the affiliation to different LHAs in vaccinated and unvaccinated children in our study sample. The sex distribution was equal in both populations and no difference was observed in the distribution of vaccinated and unvaccinated children across the two birth cohorts.

Among the 2357 ProQuad-vaccinated children, 43 varicella cases were reported, compared to 287 cases among the 912 unvaccinated children. Of these 287 varicella cases, 110 occurred between 12 and < 24 months of age, 34 occurred in children aged 24 to < 36 months, 58 in children 36 to < 48 months of age, 41 cases in children aged 48 to < 60 months and 44 cases occurred in children between 60 and < 72 months of age. Among ProQuad-vaccinated children, only two varicella cases occurred in the second and third year of life, 16 in the fourth, 9 in the fifth and 14 in the sixth. No varicella cases were observed during the first 42 days after vaccination.

### Effectiveness of a single dose of ProQuad

Varicella incidence among ProQuad-vaccinated children was 0.43 (95% CI: 0.31–0.57) per 100 person-years compared to 6.37 (95% CI: 5.66–7.16) per 100 person-years among unvaccinated children. The crude direct vaccine effectiveness was estimated at 93% (95% CI: 90–95%) and 94% (95% CI: 91–95%) when adjusted for number of visits to the FP (Table 2).

**Table 2:** Summary of vaccine effectiveness and population impact results

Vaccine effect	VE	95% CI
Direct effectiveness		
Crude	0.93	(0.90–0.95)
Adjusted	0.94	(0.91–0.95)
Total effect	0.97	(0.96–0.98)
Indirect effect	0.43	(0.35–0.50)
Overall effect	0.90	(0.89–0.91)

*VE* please define, *CI* confidence interval

### Vaccine impact: Total, indirect and overall effects

When comparing the varicella incidence among ProQuad-vaccinated children (0.43 per 100 person years, 95% CI: 0.31–0.57) with that of the historical cohort (11.87 per 100 person-years, 95% CI: 11.22–12.54), the total effect of ProQuad was estimated at 97% (95% CI: 96–98%) (Table 2).

When comparing the varicella incidence among the unvaccinated children (6.37 per 100 person-years, 95% CI: 5.66–7.16) in our study cohort to that of the historical cohort, an indirect effect of 43% (95% CI: 35–50%) was estimated (Table 2).

The overall effect was calculated by comparing the average incidence among both vaccinated and unvaccinated children in our study sample (1.2 per 100 person-years, 95% CI: 1.09–1.32) with that of the historical cohort, yielding an overall effect of 90% (95% CI: 89–91%) (Table 2).

## Discussion

To the best of our knowledge, this study was the first to measure ProQuad-specific effectiveness and the impact of a vaccination programme against varicella in children vaccinated with the quadrivalent MMRV vaccine ProQuad. The Veneto Region of Italy offered a unique possibility to conduct this study for two reasons. First, the region is covered by the Pedianet network, which includes data generated by FPs during routine patient care and stored in files using a unique, anonymised Pedianet ID that can be linked through the fiscal code to data from the Regional Immunization Database, where all vaccination data are stored. Second, the Veneto Region was the only place in Europe where the quadrivalent MMRV vaccine ProQuad was used shortly after varicella vaccination was introduced into the vaccination programme.

Values for direct effectiveness and impact were very high, with a direct vaccine effectiveness of 93% (95% CI: 90–95%), and impacts of 97% (95% CI: 96–98%), 43% (95% CI: 35–53%), and 90% (95% CI: 89–91%) for the total, indirect, and overall effects, respectively, confirming the vaccine efficacy and effectiveness reported in previous studies conducted with the monovalent vaccine Varivax, which contains the same varicella component as ProQuad [14].

When interpreting vaccine effectiveness and impact, some limitations have to be considered. When estimating direct vaccine effectiveness, we made the assumption that vaccine status was random, i.e., that the characteristics of vaccinated and unvaccinated children from the same birth cohorts did not differ. We found no differences in sex distribution or birth cohort affiliation, and adjusted for number of visits to the FP. A further assumption was equal susceptibility and an equal opportunity to be exposed to varicella (random mixing), which may not always apply when incidence rates are very low.

For the measures of vaccine impact (as measured by total, indirect, and overall effect), the study used a historical cohort to represent a pre-vaccination period [13], assuming this cohort was reasonably similar and that the method of collection of cases and susceptibility to varicella did not differ between the two time periods. While we do not know how many children in the historical cohort came from the Veneto Region, they were all registered with Pedianet FPs in Italy, and the Veneto Region was over-represented. Considering that the same software used by FPs in their routine practice (junior Bit) was used as a data collection system in both studies, differences in data collection should be limited. The historical cohort represents a population completely unexposed to varicella vaccination, as the vaccine was not yet on the Italian market, but other than this key difference, we assumed there was no other difference in susceptibility to varicella between the two populations.

Our historical cohort was based on the study sample from Nicolosi et al. [13]. However, these children were only followed for 1 year. To rule out the possibility that this was a high- or low-incidence year for varicella, we performed a sensitivity analysis using the 2006/2007 birth cohorts from a neighbouring region without a varicella vaccination programme (Lombardia) registered with Pedianet as comparator and followed them up to their 6th birthday. The total effect of 97% (95% CI: 96–98) that we obtained confirmed our results.

Based on a previously conducted pilot study in which we extracted vaccination data from the Regional Immunization Database for all children born between 2003 and 2013, we have a good picture of varicella vaccine coverage in the Veneto Region for these birth cohorts. Varicella vaccines have been available in the Veneto Region since 2004, and vaccination coverage increased rapidly, from 15% in the 2004 birth cohort to 72% in the 2005 birth cohort, who were of vaccinating age when the varicella vaccine became part of the Veneto Region’s vaccination programme. For the 2006 to 2013 birth cohorts, vaccine coverage was high and stable, between 83% and 90%.

The three vaccine impact measures (total, overall, and indirect effects) relate to the vaccination programme and depend on vaccine coverage in the population. For the total effect we compared varicella incidence in ProQuad-vaccinated children to that in the historical cohort, but this effect cannot be fully attributed to ProQuad. It is partly attributable to all varicella vaccines that were administered to children between 2004 and the end of follow-up in 2013, as it consists not only of the direct effectiveness of the vaccine that the child received but also the indirect protection associated with the herd immunity that comes with increasing vaccine coverage [12]. If the effectiveness of other varicella vaccines was lower than that of ProQuad, the level of exposure to varicella of the children vaccinated with ProQuad would be higher than expected in a hypothetical cohort of children vaccinated with ProQuad only, thus leading to an underestimation of the total effect, that can be attributed to ProQuad. On the contrary, if the effectiveness of other varicella vaccines was higher, we would have observed an overestimation of the total effect compared to a hypothetical cohort vaccinated with ProQuad only. Therefore, the estimation of direct vaccine effectiveness is the best estimate of the performance of ProQuad that is not confounded by the use of other varicella vaccines. Indirect and overall vaccine effects are not vaccine-specific; they are attributable to all vaccines used in the study period.

A general limitation of this study is that information on vaccine brand was missing for almost 60% of the children in our study sample. Therefore we performed a sensitivity analysis using the same methodology as described above, but including all vaccinated children (whatever the brand) and compared the results to the ProQuad-specific analyses. Crude direct vaccine effectiveness was 91% (95% CI: 89–93%) and 92% (95% CI: 90–93%) when adjusted for the number of visits to the FP, and the total effect of varicella vaccination with any brand was 95% (95% CI: 96–97%). These results are very similar to the ones we report here; however, as the vaccine brand was unknown in almost 60% of vaccinated children, and as we assume, based on procurement figures, that most of them were vaccinated with ProQuad, we cannot draw conclusions about the potential effectiveness of other varicella vaccines that were used in children in the Veneto Region in 2003–2013.

## Conclusion

Our study was the first to measure the ProQuad-specific effectiveness and impact of a vaccination program against varicella in ProQuad-vaccinated children. Effectiveness and impact results were very high in the Veneto Region, with an adjusted direct vaccine effectiveness of 94% (95% CI: 90–95%) and a total effect of 97% (95% CI: 96–98%), confirming the vaccine efficacy and effectiveness reported in previous studies on a single dose of the monovalent vaccine, Varivax, which contains the same varicella component as ProQuad [15, 16]. The overall effect of the vaccination program against varicella in the region was 90% (95% CI: 89–91%) and an indirect effect of 43% (95% CI: 35–53%) was also demonstrated in the unvaccinated population. For methodological reasons the Proquad-specific vaccine effectiveness was estimated for a single dose schedule, however, it is worth to point out that varicella vaccination is recommended to be given as a two dose schedule, as two vaccine doses have been shown to result in higher seroconversion rates and higher vaccine efficacy against both, severe and mild disease. The Veneto Region in Italy offered a unique opportunity to conduct this study, due to the possibility to link information from the Regional Immunization Database with data from the Pedianet database.

## Abbreviations

CI: Confidence interval
FP: Family paediatrician
LHA: Local Health Authority
MMRV: Measles, mumps, rubella, varicella
NHS: National Health Services
RR: Relative risk
VP: Vaccination programme
